# Additive Manufacturing of Ti-Based Intermetallic Alloys: A Review and Conceptualization of a Next-Generation Machine

**DOI:** 10.3390/ma14154317

**Published:** 2021-08-02

**Authors:** Thywill Cephas Dzogbewu, Willie Bouwer du Preez

**Affiliations:** 1 Department of Mechanical and Mechatronics Engineering, Faculty of Engineering, Built Environment and Information Technology, Central University of Technology, Free State, Bloemfontein 9301, South Africa; 2 Centre for Rapid Prototyping and Manufacturing, Faculty of Engineering, Built Environment and Information Technology, Central University of Technology, Free State, Bloemfontein 9301, South Africa; wdupreez@cut.ac.za

**Keywords:** TiAl, intermetallic alloys, additive manufacturing, LPBF, EBM, near-net shapes

## Abstract

TiAl-based intermetallic alloys have come to the fore as the preferred alloys for high-temperature applications. Conventional methods (casting, forging, sheet forming, extrusion, etc.) have been applied to produce TiAl intermetallic alloys. However, the inherent limitations of conventional methods do not permit the production of the TiAl alloys with intricate geometries. Additive manufacturing technologies such as electron beam melting (EBM) and laser powder bed fusion (LPBF), were used to produce TiAl alloys with complex geometries. EBM technology can produce crack-free TiAl components but lacks geometrical accuracy. LPBF technology has great geometrical precision that could be used to produce TiAl alloys with tailored complex geometries, but cannot produce crack-free TiAl components. To satisfy the current industrial requirement of producing crack-free TiAl alloys with tailored geometries, the paper proposes a new heating model for the LPBF manufacturing process. The model could maintain even temperature between the solidified and subsequent layers, reducing temperature gradients (residual stress), which could eliminate crack formation. The new conceptualized model also opens a window for in situ heat treatment of the built samples to obtain the desired TiAl (*γ*-phase) and Ti_3_Al (*α*_2_-phase) intermetallic phases for high-temperature operations. In situ heat treatment would also improve the homogeneity of the microstructure of LPBF manufactured samples.

## 1. Introduction

Although there has been ongoing research for more than a century [1,2] on manufacturing intermetallic alloys for industrial applications due to their unique high-temperature applications [2,3], it was the collaborative research launched by Oak Ridge National Laboratory (ORNL) in 1980 [2,4,5] that fast-tracked the discovery of various methods of producing intermetallic alloys for industrial applications. The ORNL project brought together over 100 research institutes to investigate the manufacturing of intermetallic alloys for industrial applications [2,4,5]. The research institutes modified and improved upon most of the methods (casting, forging, sheet forming, extrusion, etc.) of manufacturing intermetallic alloys [6]. The TiAl, NiAl and FeAl intermetallic phases were the prime focus of thr ORNL project due to their exceptional intermetallic properties [2,6,7]. Of all the intermetallic alloys developed by the ORNL project, titanium aluminide (TiAl)- and nickel aluminide (NiAl)-based intermetallic alloys have already found industrial applications or are close to commercialization [2,6]. FeAl-based alloys did not receive much attention since their critical ordering temperature is only in the range of 500–700 °C [8]. Research on NiAl-based intermetallic alloys also declined, since the NiAl alloy can only be considered a low-cost, lightweight superalloy when compared to the heavy Ni-based superalloys which are the main alloys used for high-temperature applications [9]. The decline of interest in NiAl-based alloys was exacerbated by their extremely low plasticity. Although the work of Aoki and Izumi [10] and the collaborative research project of ORNL [4] seemed to resolve the low plasticity problem of NiAl-based alloys with the addition of boron, TiAl-based alloys seem to take the center stage and have attracted much more research interest and commercialization than NiAl and FeAl-based intermetallic alloys [11,12]. 

Unlike the conventional metallic alloys whose crystal structure and physical properties depend on the ratio of the constituents of the parent materials, intermetallic alloys are formed when the resultant crystal structure and physical properties of the final material does not depend on any of the constituent materials [2]. Intermetallic alloys are a unique group of materials that are not bound by the relatively weak metallic bonds, but rather by strong ionic and covalent bonds [8]. The atoms in intermetallic alloys always take their strict positions in a crystalline lattice, resulting in the formation of a so-called ordered superlattice which is characterized by a long-range order that is stable up to a critical temperature of ordering [2,8,13]. Some intermetallic alloys maintain the ordered crystal lattice up to their melting points [1,14,15]. It is the ordered structural characteristics that make intermetallic alloys a prime choice for high-temperature applications [12]. The main properties of intermetallic alloys can be summarized as follows: high melting points, high thermal conductivity, low densities, good strength, good structural stability particularly at high temperatures, good oxidation, good wear, and good corrosion resistance at high temperatures due to the continuous formation of an adherent alumina surface layer [1,3,5,8,11]. 

The literature reveals that the infrastructure and knowledge base of producing TiAl-based intermetallic alloys via the conventional methods (casting, forging, sheet forming, extrusion, wire assembly, etc.) [6,16] are very mature, and their technological maturity has been proven by the recent application of the alloy in the General Electric GEnX jet engine [17]. It is reported that replacing the heavyweight Ni-based superalloys in jet engines, turbine engines, aero-engines etc. with TiAl-based intermetallic alloys could reduce the structural weight of high-performance engines by 20–30% [18,19,20]. Unfortunately, the low susceptibility to plastic deformation and a high tendency for brittle cracking [6] of TiAl-based intermetallic alloys have made it very challenging to use the conventional methods to produce near-net shape components that would enhance the performance and efficiency of TiAl-based intermetallic alloy components [2,17]. 

It has been suggested that intermetallic alloys could contribute significantly to the production of high-tech devices of heat exchangers, microreactors, and microactuators, due to their high temperature properties if they could be manufactured in the form of lattice structures and tapes [21,22,23]. Compared to the properties of the conventional materials that are used for the production of microsystems, intermetallic alloys exhibit attractive properties such as high working temperatures (up to 1300 °C), high strength, relatively high ductility and fracture toughness, high oxidation and corrosion resistance, and high thermal stability, which make them the right choice for microsystems applications [24,25,26]. However, the inherent limitations of the conventional manufacturing systems to produce intricate shapes (e.g., back tapers, intricate cooling channels, customized porous structures, special lattices, hollow structures, etc.) [27,28] make the production of such microsystems using the TiAl-based intermetallic alloys very challenging. Meanwhile, it is well documented that to improve the energy efficiency and power of highly engineered products such as the aircraft engines, industrial gas turbines, automotive parts and microsystems, near-net shapes that would enhance the geometrical, technical and functional properties of the components are paramount [29,30]. Hence, to satisfy the industrial requirements of producing TiAl-based intermetallic alloys with geometrical, technical, and functional components of complex geometries, alternative manufacturing routes need to be identified [20]. 

Fortunately, the rapid development in manufacturing technologies and the emergence of additive manufacturing (AM) has provided an opportunity for possible manufacturing of intermetallic alloys according to the current industrial requirement of intricate shapes [29]. Manufacturing TiAl-based alloys with near-net shapes would enhance the operational performance and the widespread engineering applications of the TiAl-based intermetallic alloys. AM technology is currently considered a renaissance of the manufacturing industry, providing it with superior manufacturing capabilities [28]. AM technology could be used to produced three-dimensional (3D) printed parts with intricate geometrical configurations [31]. It is considered a monolithic manufacturing process that gives the manufacturing engineer freedom of design to produce near-net shapes for tailored engineering applications [12,28]. The unique capabilities of AM manufacturing technology for producing near-net shapes of complex geometries for tailored applications have triggered intensive academic and industrial research on manufacturing TiAl-based intermetallic alloys with intricate geometries via AM technology. 

General Electric (GE) [16] uses AM technology to produce aircraft engine components that power the Boeing 787 and 747–8 engines as an alternative to the conventional method (gravity casting). Using AM technology to produce engine components with near-net shapes has led to a 50% reduction in noise, a 20% reduction in fuel consumption, and an 80% reduction in NOx emissions, compared with previous engines in a similar class. The propulsion efficiency of the Boeing 787 and 747–8 engines has greatly increased, and over 40,000 TiAl-based intermetallic alloy components have been manufactured for aerospace applications via AM manufacturing technology. Obviously, using AM technology to produce the TiAl-based intermetallic alloy components would enhance the performance and efficiency of the alloy for high-temperature applications. This review outlines the extent to which AM technology is able to produce TiAl intermetallic alloys with intricate geometrical characteristics to enhance their technical and functional performance. The paper also presents a schematic description of improving the AM manufacturing systems to produce crack-free TiAl-based alloys. 

## 2. TiAl Intermetallic Alloy Phases 

**Figure 1:**
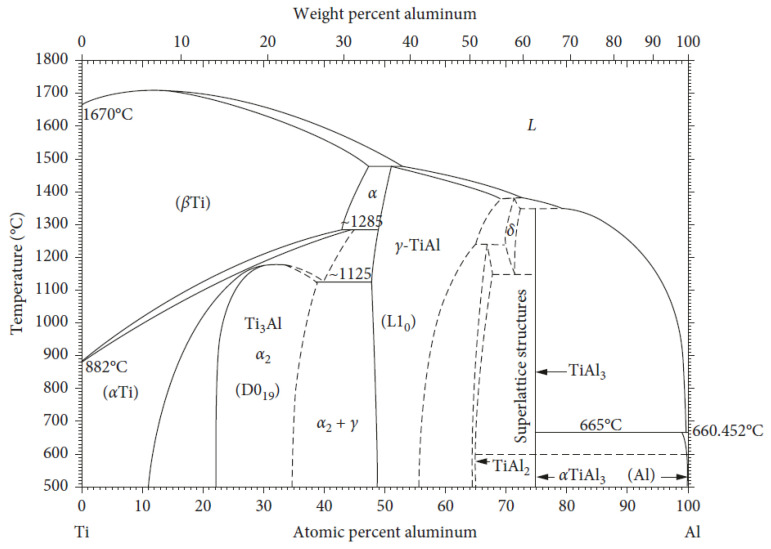
TiAl phase diagram [32].

It can be observed from the TiAl phase diagram (Figure 1) that the solidification of the TiAl alloy phases is strongly composition-dependent [11,32]. 

The solidification of the TiAl alloy can occur in a single-phase: TiAl_3_, *γ*(TiAl), or two phases: γ(TiAl) + $\alpha_{2}\left({\mathrm{Ti}}_{3}\mathrm{Al}\right)$. The alloy solidifies in a single phase when the Al composition exceeds 50–56 at.% [Ti(50-56)Al] and in a double phase when the composition is between 44 and 49 at.% [Ti(44-49)Al] [33,34]. The TiAl_3_ phase of the alloy does not have much engineering relevance [35]. The attention of industrial practitioners and academic researchers was drawn to the *γ*(TiAl) single-phase alloy, due to its outstanding resistance to environmental attack (hydrogen absorption and oxidation). However, it was later discovered that the *γ*(TiAl) single-phase alloy demonstrated poor fracture toughness and ductility at room temperature [33,34]. This limitation has led to a downward revision of their research trajectory by both academic researchers and industrial practitioners, probably due to the outstanding advantages offered by the two-phase γ(TiAl) + $\alpha_{2}\left({\mathrm{Ti}}_{3}\mathrm{Al}\right)$ alloy. The two phase TiAl-based alloy can maintain its ordered crystal lattice until its melting point, which makes it ideal for high-temperature applications. As a result, there has been ongoing research to enhance the structural applications of the two-phase alloy [19,20,33,34]. The aim was to achieve balanced mechanical properties (toughness, ductility, creep strength, and tensile strength) via improved alloy chemistry, manufacturing process, and microstructural control [33,34]. Alloys with various elemental compositions from three different designated groups have been vigorously investigated to improve on the structural applications of the two-phase γ(TiAl) + $\alpha_{2}\left({\mathrm{Ti}}_{3}\mathrm{Al}\right)$ Ti-(44-49)Al alloy. The formulations of the compositions of the designated groups are presented as: Ti-(44-49)Al-(1-3)X_1_-(1-4)X_2_-(0.1-1)X_3_ (X_1_ = V, Cr, Mn; X_2_ = Nb, Ta, W, Mo; X_3_ = Si, C, B, N, P, Se, Te, Ni, Mo, Fe). It was reported that the room temperature ductility of the two-phase TiAl alloy nearly doubles by adding the X_1_ alloying elements. The oxidation resistance and solid solution strengthening of the two-phase TiAl alloy was greatly improved by the X_2_ elements. The creep resistance, oxidation resistance, and ductility of the alloy was greatly improved by the X_3_ alloying elements [19,20,33,34]. The two-phase γ(TiAl) + $\alpha_{2}\left({\mathrm{Ti}}_{3}\mathrm{Al}\right)$ Ti-(44-49)Al alloys are currently considered to be the most suitable candidates for the replacement of Ni-base superalloys for high-temperature applications [36,37]. For the two-phase TiAl alloys, the γ(TiAl) serves as the matrix base and the $\alpha_{2}\left({\mathrm{Ti}}_{3}\mathrm{Al}\right)$ is distributed in the matrix. The nature of the microstructure of the material is important as it dictates the resulting mechanical properties of the alloy both at high temperature and room temperature [35]. The TiAl alloy exhibits three unique microstructures (fully or near lamellar, gamma or near gamma, and duplex microstructures) based on its composition, manufacturing process, and thermal treatment [38,39].

✓ The fully lamellar or nearly lamellar microstructure consists of the TiAl (γ-phase) and a small volume fraction of Ti_3_Al (α_2_-phase).
✓ Near-gamma: the near-gamma alloy consists of a gamma (γ) grain microstructure with a moderate alpha grain.
✓ Duplex: the duplex microstructure consists of gamma (γ) grains, B2 phase, and γ/B2 lamellar colonies.

The fully lamellar and nearly fully lamellar microstructures consisting of TiAl (γ-phase) and a small volume fraction of Ti_3_Al (α_2_-phase) demonstrated high oxidation resistance, high fracture toughness, high creep, and crack propagation resistance, which make such TiAl alloy phases ideal for high-temperature applications in comparison to the duplex and gamma microstructures [38,39]. Although the duplex and the gamma microstructures demonstrated high strength and some ductility, they exhibited low fatigue strength, low fracture toughness, and poor creep resistance, the latter being properties required for most engineering applications at high temperatures [38,39]. 

Since the performance of a material does not depend only on its mechanical properties but also on its geometrical configurations [12,28,40], using the emerging AM manufacturing technology to produce the two-phase γ(TiAl) + $\alpha_{2}\left({\mathrm{Ti}}_{3}\mathrm{Al}\right)$ Ti-(44-49)Al alloy with near-net shapes would certainly improve its technical and functional performance.

## 3. Metal Additive Manufacturing 

Additive manufacturing (AM) could be described as the process of producing 3D components from metallic materials (powder feedstocks, wires, sheet forms, etc.) layer-by-layer, as opposed to the subtractive approach applied in conventional manufacturing methods [41]. The American Society for Testing and Materials (ASTM) international committee F42 on AM technologies [42] classified AM manufacturing technologies into seven categories, namely: powder bed fusion, directed energy deposition, sheet lamination, photopolymer vat, material extrusion, material jetting, and binder jetting. Powder bed fusion, directed energy deposition, and sheet lamination belong to metal additive manufacturing (MAM), which means the powder feedstocks are metallic materials such as metallic powder particles [43]. Powder bed fusion manufacturing systems are typically employed to produce intricate geometries requiring high resolution and rigorous build accuracy [44], while directed energy deposition systems are commonly applied to repair and refurbish metal parts and for large-scale manufacturing [43,45]. Sheet lamination systems have the capability of joining dissimilar metals to produce components with some specific properties [43]. The outstanding capability of powder bed fusion (PBF) systems of producing 3D components of complex geometries with high resolution and rigorous build accuracy have made them the preferred choice for manufacturing intricate 3D objects with tailored geomaterial configurations [28]. 

The PBF manufacturing process is a real paradigm shift from the traditional subtractive manufacturing methods to the realization of manufacturing 3D components with intricate geometrical characteristics. It is a layer-by-layer monolithic eco-design topology optimization manufacturing technology that permits the manufacturing of 3D components of intricate geometries according to the technical, functional, and geometrical dimensions of the required/intended applications [27]. Using the versatility of PBF systems to manufacture TiAl-based intermetallic alloys with intricate shapes, such as honeycomb/lattice structures, which are ultralightweight metamaterials with high specific strength, high specific rigidity, high durability, high energy absorption rates, and thermal protection, would enhance the high-temperature applications of the TiAl-based intermetallic alloys [28]. Using the conventional methods (casting, forging, sheet forming, perforated/slotted, sheet folding, extrusion, wire assembly etc.) to manufacture components with intricate geometries made it very challenging, if not impossible, to produce periodic structures of TiAl-based alloys with complex, tailored geometrical configurations [46]. The classical methods of producing intricate shapes required multiple processing steps of perforating, folding from metal sheets, chiselling, etc. These steps are time consuming and permitted only the manufacturing of simple geometries. 

Using the PBF monolithic manufacturing process to produce TiAl-based alloys with complex tailored geometrical configurations would eliminate the machining and joining steps, as well as the tooling/reworking processes employed in the conventional methods of manufacturing. These advantages of using PBF technology would reduce the time to market production and the buy-to-fly ratio, owing to a low material waste factor, creation of supplementary functions such as cooling channels, and weight savings due to the tailored geometries [47]. These unique characteristics have demonstrated that PBF technology could be a cost-effective method for producing TiAl-based intermetallic alloys according to the current industrial requirements. In addition, it is reported that the unique intermetallic structural benefits of TiAl-based alloys could be lost when using conventional manufacturing techniques to produce TiAl-based intermetallic alloys [14]. It is therefore paramount to use the versatile monolithic additive manufacturing strategy of the PBF process to produce TiAl-based intermetallic alloys that maintain the ordered superlattice structure that gives the alloy its high temperature properties. The PBF manufacturing systems comprise of laser powder bed fusion (LPBF) and electron beam melting (EBM) machines. LPBF manufacturing systems use laser beams to selectively melt the metallic feedstocks, while EBM manufacturing systems use electron beams to selectively melt the metallic feedstocks according to the CAD (computer-aided design) model. 

## 4. Electron Beam Melting of TiAl-Based Alloys 

**Figure 2:**
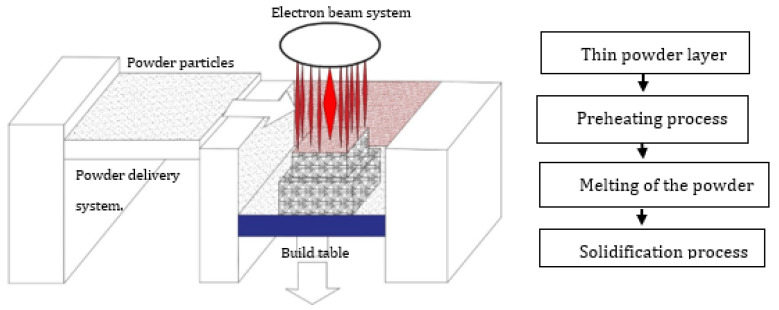
Schematic representation of the EBM manufacturing system.

EBM systems are comprised of the electron beam compartment and the build chamber [48]. As schematically represented in Figure 2, the TiAl metallic powder feedstock is delivered to the powder bed by hoppers, and a rake spreads the TiAl powder feedstock on the build table during the layering process. A defocused electron beam (electron beam of lower power) preheats or sinters the TiAl powder feedstock according to the 3D CAD model. Then, a focused electron beam (full electron beam power) melts the preheated powder layer. The higher-power electron beam melts the powder, and the molten pool solidifies into a fully dense layer with a fine-scale microstructure [49,50]. The build table is lowered equivalent to the powder layer thickness. A new TiAl powder is delivered from the hoppers and spread by the rake to ensure even distribution of the powder on the build table. The new powder layer is then preheated by the lower power electron beam and the whole process is repeated. These four basic processes of powder spreading, pre-heating, melting and solidification are repeated until the 3D object is completely fabricated according to the CAD design [49,51].

Cormier et al. [52] used the EBM system to produced bulk intermetallic Ti-47Al-2Cr-2Nb components with pre-alloyed and elemental blended powders mixed in appropriate ratios. Optical and electron microscopic observations of the microstructures revealed that the samples produced with the pre-alloyed TiAl powder demonstrated a lamellar *α*_2_ + *γ* TiAl microstructure, while the microstructure of the elemental blended powders was TiAl_3_, which does not have much engineering significance [35]. Murr et al. [53] conducted a similar experiment via the EBM system using a pre-alloyed powder of nominal composition Ti–47Al-2Nb-2Cr (at.%) and precursor powder in appropriate ratios. The samples produced with the pre-alloyed powder feedstock revealed a γTiAl grain structure with a lamellar γ/$\alpha_{2}$ colony structure. The microstructure of the samples produced with precursor powder feedstock was largely $\alpha_{2}$-phase rich (Ti_3_Al) with un-melted aluminum and titanium powder particles. 

The formation of the TiAl (γ-phase) and Ti_3_Al (*α*_2_-phase) phases of the TiAl components produced by the EBM systems demonstrated that EBM systems could be used to produce TiAl intermetallic alloy parts with near-net shapes for high temperature engineering applications. Using EBM manufacturing technology to produce TiAl intermetallic alloys with intricate geometries which would meet the current industrial requirements would ensure the widespread industrial application of TiAl intermetallic alloys. A thorough review of the literature reveals that optimized processing conditions have already been achieved for producing sound and crack-free TiAl-based alloy components using EBM manufacturing processes [47,53,54,55]. The outstanding capability of EBM manufacturing systems to produce crack-free TiAl components is attributed to its preheating system prior to the melting of the the metallic feedstock. The preheating system maintains the temperature in the build chamber in the order of 1000 °C [37,53], lowering the thermal gradient and the residual stress during the building process, which enables the production of crack-free 3D structures using TiAl-based alloys. 

Although published studies have demonstrated the outstanding capability of the EBM manufacturing process to produce crack-free TiAl components with the desired intermetallic phases, the literature also suggests that EBM manufacturing systems demonstrate a lack of geometrical precision due to the melt pool dimensions [53,54,56]. The lack of geometrical precision demonstrated by EBM systems requires larger machining allowances. Due to the complex geometry of the final 3D components, post-processing activities become very complicated [12]. Trying to machine the EBM manufactured components to the final precise dimensions would also result in waste of the material, which undermines one of the main benefits of PBF manufacturing technology to save material [29]. The inherent limitation of the EBM manufacturing system makes it very difficult to achieve the objective of implementing PBF manufacturing technology to produce tailored TiAl components with complex geometries such as cooling channels, since machining intricate shapes can be extremely difficult, or even impossible in some cases. This drawback of EBM manufacturing technology has triggered the desire of researchers and industry practitioners to experiment with other PBF technologies such as the LPBF manufacturing process, which is perceived to have high dimensional accuracy [28,57,58]. The preheating strategy of the EBM systems also increases the loss of Al content in the final built parts as compared to LPBF manufactured components [33,37,54]. 

## 5. Laser Powder Bed Fusion of TiAl-Based Alloys 

LPBF manufacturing systems have been considered the most versatile among the PBF manufacturing systems due to their smaller beam size (small melt pool dimensions as compared to EBM), which gives LPBF manufacturing systems the advantage of geometrical precision as compared to EBM [30,56,57,58]. The high degree of dimensional accuracy due to the smaller beam size reduces the burden of post-processing activities. As a result, it has been used extensively for manufacturing several biomedical and engineering components using titanium and steel-based alloys with great dimensional accuracy [58]. The literature reveals that, compared to EBM manufacturing systems, LPBF technology has not been used extensively to manufacture TiAl-based alloys [12,54]. The production of TiAl-based intermetallic alloys via the LPBF manufacturing process only started about a decade ago [59]. Unfortunately, the pioneers could not use the LPBF manufacturing process to produce crack-free TiAl samples [54,59,60,61]. The high rate of heating and cooling (10^4^–10^6^ K/s) [62] during the LPBF manufacturing process, which results in the build-up of residual stress, was responsible for the inability to produce crack-free TiAl near-net components [28,37]. A host of researchers tried to determine the optimum process parameters that could be used to produce crack-free TiAl near-net-shapes, but to no avail [54,59,60,61,63]. These attempts were based on the premise that there are more than 50 processing parameters [64] that influence melt pool geometry during the LPBF process and the appropriate combinations of these parameters might help to overcome the cracking effect. 

**Figure 3:**
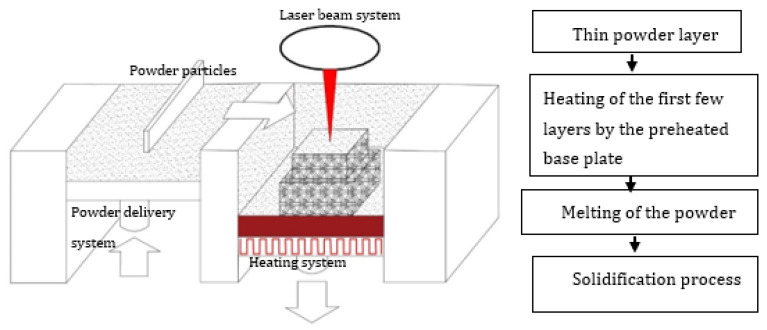
Schematic representation of the LPBF manufacturing system.

Vilaro et al. [59] changed the solidification front to induce a smaller temperature gradient by a combination of the process parameters of slow scanning speed and wider beam diameter (scanning speed 0.02 m/s, laser beam diameter 380 μm, laser power 60–250 W). The thermal conditions (temperature gradient—G, cooling rate—T, solidification rate—Z) were optimized by changing the solidification behavior from dendritic, to cellular, up to planar front growth. Thus, an increase in the G/Z ratio progressively changes the solidification behavior, whereas the G/Z ratio determines the microstructure characteristics. A careful combination of the process parameters could lead to the production of crack-free TiAl-based alloy components with intricate geometries by slowing down the cooling rate. The optimum process parameters cause the molten pool to take a longer time to solidify at a lower cooling rate. The effort of Vilaro et al. [59] was only able to reduce the cracking effect, but could not suppress it completely. Vilaro et al. [59] reported that the cracks began from the interface of the solidified layers, and propagated along the building direction Z. The authors maintained the building substrate at 500 °C (Figure 3) throughout the experiments to relieve the material of residual stress while the building process continued. This strategy could not prevent the cracking of the TiAl build parts because the LPBF manufacturing process works by lowering the base plate a distance equivalent to the powder layer thickness each time a layer is completed. Because a new layer of powder is delivered onto the powder bed and the manufacturing process continues, for a large multiple layer component, the effect of keeping only the base plate at 500 °C becomes ineffective for preventing crack formation. In reality, the approach of holding the base plate at a high temperature has the tendency of inducing a high thermal gradient between the base plate section and the top section of the built component. The high temperature gradient introduces high residual stress, which could cause the sample to crack.

Gussone et al. [65] focused on investigating the possibility of using a preheating system termed intrinsic heat-treatment to produce crack-free Ti-44.8Al-6Nb-1.0Mo-0.1B (at.%) intermetallic alloy components via LPBF. A heating cycle was introduced between the new layer and the already solidified layers during the manufacturing process. “The successive heating of the solidified layers as new powder layers were deposited and melted is termed intrinsic heat treatment” [12]. In addition to the intrinsic heat treatment, the base plate was held at 800 °C (Figure 3) during the manufacturing process. The TiAl components were manufactured at a scanning speed of 0.45 m/s, a laser power of 80 W, and a powder layer thickness of 30 µm. However, the intrinsic heat treatment could not eliminate the crack formation. It was reported that a tension crack evolved from the side of the specimen towards the middle, which implied that there a thermal gradient developed between the outer perimeter and the middle section of the sample. Hence, intrinsic heat treatment could not prevent the rapid heating and cooling process inherent in the LPBF manufacturing process. The intrinsic heating process led to the production of dissimilar microstructural features in different areas of the samples. The researchers found that the microstructures of the samples were improved using hot isostatic pressing (HIP). HIP enabled the production of the ($\alpha_{2}/\gamma$) lamellar microstructure.

Loeber et al. [54] conducted a direct comparison between the EBM and LPBF manufacturing processes. These authors used the EBM and LPBF manufacturing process to produce Ti-48Al-2Cr-2Nb samples. Microstructural analysis revealed that the LPBF samples did not show any clear microstructure under the SEM-SE (secondary electron) contrast—thus no clear statement could be made of the microstructure of the as-built LPBF samples. A fine lamellar microstructure was observed after heat treating the samples at 1400 °C for 2 h. The LPBF samples had elastic modulus values of 50 ± 13 GPa, which were about one third lower than what is normally reported for TiAl alloys manufactured using the EBM and the conventional manufacturing methods [55]. The low elastic modulus of the LPBF samples was attributed to the presence of cracks during the LPBF manufacturing process. Obviously, the attempt by these authors to produce crack-free TiAl-based intermetallic alloys via the LPBF systems was not successful.

**Figure 4:**
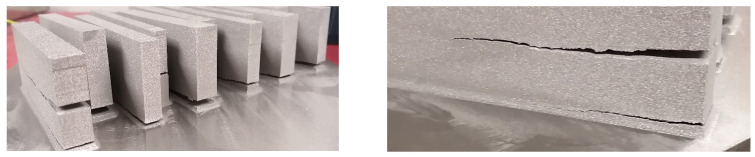
Destructiveness of residual stress due to the temperature gradient [76] (Courtesy of CRPM).

Many preheating techniques were introduced (Figure 3) (scanning laser beam [66,67,68], IR heaters [69,70], fast and second defocused laser beams [71], baseplate induction circuits [61,65,72], and substrate resistive heating [73,74]) to overcome the cracking effect of TiAl components manufactured via LPBF. The preheating techniques were focused on heating the base plate to alleviate the cracking effect during the LPBF manufacturing process. However, as presented in Figure 2, the EBM preheating system is designed to keep the powder bed at a high temperature (about 1000 °C) [37,55] throughout the manufacturing process prior to the melting of the powder feedstock by the lower power electron beams, as opposed to the LPBF pre-heating system which is mainly focused on preheating of the base plate (Figure 3). The base plate normally acts as a heat sink, which could serve as a crack initiation point due to the high thermal gradients and thermal stresses at the bottom of the samples [59]. As a result, many researchers and industry practitioners have focused on preheating the base plate in an attempt to avoid the cracking effect. This analogy sounds very logical, because most of the cracking during the LPBF manufacturing process starts from the base plates (Figure 4). However, the building of large TiAl components requires keeping every layer of the powder bed at a high temperature to avoid the development of a high thermal gradient between the solidified layers and the new layers. Heating only the base plate provides a narrow window of producing only a few crack-free layers at the base plate level. As the building process continues and the manufactured samples increase in size, the effectiveness of heating only the base plate diminishes, and the samples begin to crack from the interface between the layers, the outer perimeter, and the middle section of the samples, as previously reported by Vilaro et al. [59] and Gussone et al. [65] (Figure 4). It is obvious that the industrial panorama of producing TiAl-based intermetallic alloy components of intricate shapes with great geometrical accuracy is in high demand [16,75]. Therefore, there is an urgent need for further research into the possibility of producing crack-free intricate geometries of TiAl-based alloy parts with great dimensional precision for high-temperature applications via LPBF.

## 6. The Proposed Next Generation LPBF Manufacturing Systems

**Figure 5:**
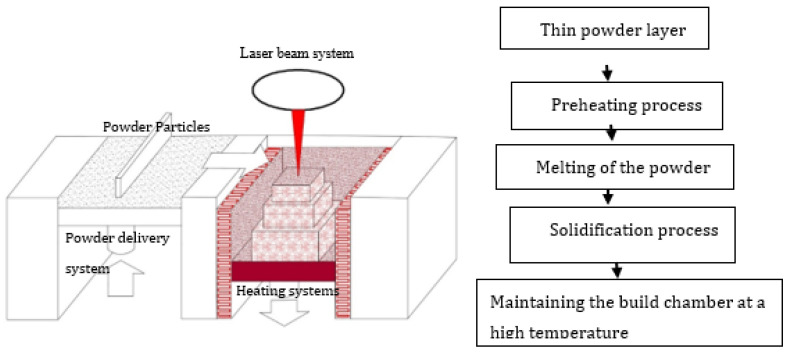
Schematic representation of the conceptualized LPBF manufacturing system.

The authors discussed in the previous section focused on preheating only the base plate at 500 °C and 800 °C and reported on crack formation in the final built parts [37,59,65]. The next generation of LPBF systems, which could prevent the high thermal gradient and the subsequent residual stress build up during the building process to avoid crack formation in the final 3D component, is schematically depicted in Figure 5. The proposed LPBF system focuses on reducing the temperature gradient between the solidified layers and the new layers by keeping the entire building chamber at a temperature which ensures even cooling during and after the building process, unlike the previous LPBF system which focused only on pre-heating the base plate. The layer-wise manufacturing strategy of the LPBF process ensures that the build platform is lowered one step according to the powder layer thickness after a layer is built. The current proposed system (Figure 5) would ensure that the temperature of the already built part on the built platform is at a temperature that would enable a slow cooling rate during and after the entire build process. Such an LPBF manufacturing process would not favor the development of a high thermal gradient. Since the temperature gradient between the bottom part of the sample and the upper parts is reduced, there is a greater possibility of producing crack-free TiAl samples with tailored geometrical configurations.

Recently, Caprio et al. [37] experimented with the conceptualized next generation LPBF system presented in Figure 5. The authors maintained the preheating temperature of the base plate and the previously solidified layers at 800 °C throughout the LPBF process. A 50-layer 3D component was manufactured. Their analysis revealed that there were pores (holes) within the samples. Keeping the base plate and the whole build chamber at 800 °C might have contributed significantly to the pore formation. The high preheating temperature of the powder bed prior to the melting process could have enhanced the absorption of the laser radiation of the powder bed, which could have resulted in keyhole formation [77,78]. Keyhole formation is a known phenomenon during the LPBF process, where the laser energy released onto the powder bed is enough to melt the powder completely, and with the excess energy ‘drilling’ into the molten pool and entrapping gas in the wake of the laser beam during the solidification process, the result is the formation of holes (keyhole effect) in the solidified built part [79]. The keyhole phenomenon can be avoided by careful study of the optimum process parameters that are used to melt the TiAl powder feedstock at each selected preheating temperature. Determining the optimum process parameters, which could compensate for the already preheated powder bed before the melting of the powder, could possibly provide the solution of using the LPBF system to produce intricate, crack-free TiAl components of high dimensional precision for high-temperature applications. Caprio et al. [37] did not characterize the TiAl phases of the samples after keeping the whole manufacturing system at 800 °C. However, the intrinsic heat-treatment of Gussone et al. [65] at 800 °C resulted in the production of dissimilar microstructural features in different areas within the samples. Graded and metastable microstructures were present in the building direction of the samples. It was also reported that the samples close to the preheated base plate recorded the highest Al loss and exhibited acicular or platelet structures ($\alpha_{2}$ within β/B2). The amount of thermodynamically generated γ-phase was greatly reduced. The top surface of the sample presented a globular shaped ultrafine lamellar ($\alpha_{2}/\gamma$) microstructure. It could be inferred that keeping the whole building system at elevated temperatures would have a significant effect on the resultant microstructures.

Obviously, there is a need for further research on regulating the temperature of the heating system (preheating system) to produce only the desired TiAl (γ-phase) and Ti_3_Al (*α*_2_-phase) intermetallic phases for high-temperature applications. Further research on in situ monitoring could help determine the optimum process parameters and the preheating temperatures that could permit the manufacturing of crack-free TiAl alloy components of intricate geometries with the TiAl (γ-phase) and Ti_3_Al (*α*_2_-phase) intermetallic phases via LPBF.

**Figure 6:**
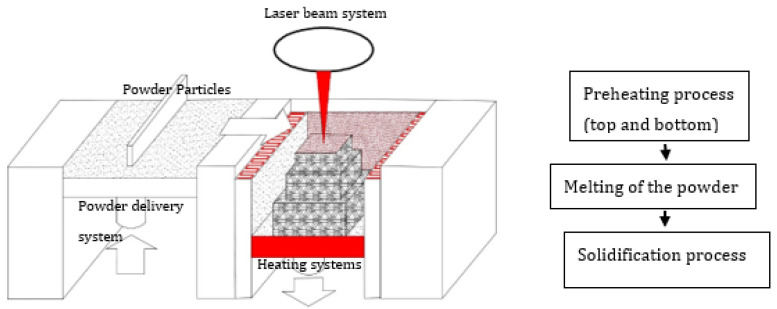
Schematic representation of the LPBF manufacturing system—preheating process (top and bottom).

The experimental investigation of Wilkes et al. [71] and the modelling results of Aggarangsi et al. [80] revealed that preheating or remelting strategies of the top layer (Figure 6) by a second defocused laser beam could produce crack-free LPBF samples of small dissensions—about 10 layers. The preheating or remelting implies double scanning, which would notably increase production time due to the double scanning of the powder bed. Due to the rapid heating and cooling, as the samples size increases, a temperature gradient would develop between the base plate section and the top section of the sample. Such a high temperature gradient could initiate tension cracks, as reported by Gussone et al. [65]. Compared to the EBM system of pre-heating which could be described as a double scanning system, the current conceptualized LPBF system is a single scanning strategy, which would reduce the time spent on manufacturing crack-free samples of intricate shapes with homogenous microstructures. Keeping the entire build chamber at a high temperature, (500 °C–800 °C) as described in the conceptualized LPBF system (Figure 5), would enable a constant preheating throughout the whole LPBF manufacturing process, as already attempted by Caprio et al. [37]. The cooling rate after the building process would also be controlled to prevent any crack formation due to rapid cooling. Such an approach would also open a window for the in situ heat treatment of the build samples to obtain the desired TiAl (γ-phase) and Ti_3_Al (*α*_2_-phase) intermetallic phases for high temperature operations. In situ heat treatment would also prevent the formation of inhomogeneous microstructures, as it is well documented that the LPBF built parts normally present anisotropic microstructures [28,41,81].

Based on the current conceptualized LPBF system and the reported experimental investigations documented so far (summarized in Table 1), it could be envisaged that the goal of manufacturing crack-free and pore-free TiAl-based 3D structures of intricate geometry with dimensional accuracy for industrial applications is very near, as the LPBF technology is gradually attaining maturity.

## 7. Conclusions

The conventional methods used to manufacture TiAl-based intermetallic alloys for high-temperature applications lack the capacity to produce intricate tailored geometries. The quest to produce TiAl components with intricate geometries to satisfy current industrial requirements has led to the production of TiAl-based alloys using emerging AM manufacturing technologies. EBM manufacturing technology is able to produce a TiAl alloy with the required TiAl (*γ*-phase) and Ti_3_Al (*α*_2_-phase) phases for high-temperature applications, but lacks geometrical precision. LPBF manufacturing systems have the potential to manufacture TiAl intermetallic alloy parts of intricate geometries with great dimensional accuracy. However, the development of high thermal gradients during the LPBF manufacturing process leads to a cracking effect during the production of TiAl intermetallic alloys via LPBF systems. A next-generation conceptualized model of the LPBF process has been proposed. It is envisaged that the model could be used to produce crack-free 3D components of intricate geometry with the required TiAl phases (TiAl (*γ*-phase) and Ti_3_Al (*α*_2_-phase)) for high-temperature applications.

## Figures and Tables

**Table 1:** Conventional and PBF manufacturing methods.

Manufacturing Methods		Disadvantages	Advantages
Conventional	Casting	➢ Cannot produced complex shapes of tailored geometries. ➢ Multiply assembly steps. ➢ Wastage of materials. ➢ Possible disruption of the ordered superlattice structure [1,14,15,46].	The infrastructure and knowledge base of producing TiAl-based intermetallic alloys via conventional methods is very mature, resulting in the mass production of simple shapes [6,16,17].
	Forging		
	Sheet forming		
	Extrusion		
Powder bed fusion	EBM	➢ Lack of geometrical accuracy of manufactured 3D components. ➢ Large machining allowances [27,28].	Production of 3D components of less intricate shapes with the required TiAl (*γ*-phase) and Ti_3_Al (*α*_2_-phase) intermetallic phases for high temperature operations [27,28].
	LPBF	Production of non-crack-free 3D components [28,57,58].	3D components of intricate geometries with high resolution and rigorous build accuracy [30,56,57,58].
The proposed LPBF manufacturing heating system	Figure 5	Optimum process parameters not yet determined.	Possible production of 3D components of intricate geometries according to the technical, functional, and geometrical dimensions of the required/intended applications.

## Data Availability

Data sharing is not applicable to this article.
